# Serum retinol-binding protein 4 levels are elevated in non-alcoholic fatty liver disease

**DOI:** 10.1111/j.1365-2265.2007.03072.x

**Published:** 2008-04-01

**Authors:** J A Seo, N H Kim, S Y Park, H Y Kim, O H Ryu, K W Lee, J Lee, D L Kim, K M Choi, S H Baik, D S Choi, S G Kim

**Affiliations:** * Division of Endocrinology and Metabolism, Department of Internal Medicine, College of Medicine, Korea University Korea; † Department of Biostatistics, College of Medicine, Korea University Korea; ‡ Department of Endocrinology, School of Medicine, Konkuk University Seoul, Korea

## Abstract

**Objective:**

Retinol-binding protein 4 (RBP4) is a recently identified adipokine that is elevated in the serum in several insulin-resistant states. We investigated the relationship between non-alcoholic fatty liver disease (NAFLD) and serum RBP4 in nondiabetic adults.

**Methods:**

One hundred and fifty-nine nondiabetic, non-alcoholic subjects (95 males and 64 females) participated in this study. Division of subjects into a NAFLD group (*n* = 73; 45 males and 28 females) or a normal group (*n* = 86; 50 males and 36 females) was based on the presence of fatty liver disease determined by sonography.

**Results:**

Serum RBP4 levels in the NAFLD group were significantly higher than those in the normal group (62·8 ± 16·0 mg/l *vs.* 51·7 ± 14·6 mg/l, *P* < 0·0001). Multiple logistic regression analysis revealed that the RBP4 level was an independent factor associated with NAFLD (*P* = 0·0042). In addition, serum RBP4 levels were positively correlated with serum alanine aminotransferase (ALT), aspartate aminotransferase (AST) and γ-glutamyltranspeptidase (GGT) levels. The significant association between serum RBP4 and GGT levels remained even after adjusting for age, gender, body mass index, the homeostasis model of assessment (HOMA) value and the presence of NAFLD (*r* = 0·3097, *P* = 0·0002).

**Conclusion:**

Serum RBP4 levels are significantly associated with NAFLD and liver enzymes.

## Introduction

Non-alcoholic fatty liver disease (NAFLD) is a major liver disease found throughout the world. It has a broad spectrum of manifestations ranging from simple steatosis to non-alcoholic steatohepatitis and cirrhosis.^1^,^2^ Obesity, hyperglycaemia, type 2 diabetes (T2DM) and hypertriglycaeridaemia are known risk factors for the development of NAFLD. NAFLD is therefore regarded as a manifestation of the metabolic (or insulin resistance) syndrome.^3^–^6^ Although its clinical association seems to be well established, the pathogenesis of NAFLD has not been fully elucidated. As obesity is the most important risk factor for insulin resistance, adipose tissue and adipokines have become the focus of study for NAFLD. Adiponectin is a representative adipokine that is associated with NAFLD. We have previously reported that serum adiponectin levels are significantly reduced in NAFLD subjects compared to healthy controls.^7^ Moreover, recombinant adiponectin improves insulin resistance and fatty liver disease by neutralizing tumour necrosis factor-α (TNF-α) activity in mice.^8^

Retinol-binding protein 4 (RBP4) is a newly identified adipokine that is elevated in the serum in several insulin-resistant states.^9^ Yang *et al.* have reported that elevation of serum RBP4 causes systemic insulin resistance, and reduction of serum RBP4 improves insulin action in mice.^9^ Likewise, serum RBP4 levels correlated with the magnitude of insulin resistance in human subjects with obesity, impaired glucose tolerance or T2DM.^10^ Furthermore, RBP4 levels have been shown to decrease in subjects in whom insulin resistance improved after exercise training.^10^ The close relationship between NAFLD and insulin resistance or obesity suggests that this new adipokine may play a role in the pathogenesis of NAFLD. Therefore, this study was designed to investigate whether there is a relationship between NAFLD and serum RBP4 levels in nondiabetic adults.

## Methods

### Subjects and measurement

The participants in this study were recruited from individuals who were self-referred for a routine health check-up at Korea University Ansan Hospital. The medical health check-up provided by the hospital included an abdominal ultrasound. We enrolled patients found to have a fatty liver. Thereafter, we selected subjects who did not have a fatty liver, matched for age and gender, as controls. Subjects were excluded from this study if they met any of the following criteria: (i) alcohol drinking >140 g/week; (ii) a positive test for hepatitis B surface antigen or hepatitis C antibody; (iii) evidence of toxic hepatitis; (iv) presence of previously diagnosed diabetes or fasting glucose ≥ 7·0 mmol/l; (v) other endocrine disease (e.g. thyroid dysfunction); (vi) known liver or renal dysfunction; or (vii) age < 20 or > 80 years. All subjects denied taking drugs known to promote fatty liver disease or to cause insulin resistance over the past 3 months. As a result of the baseline investigations including the abdominal ultrasound, 73 subjects (45 males and 28 females) with fatty liver and 86 healthy subjects (50 males and 36 females) matched for age and gender as normal controls were enrolled in this study. Informed consent was obtained from all subjects before enrolment in the study, which was approved by the ethics committee of the institution. Clinical data such as age, gender, height, weight and body mass index were recorded. Systolic and diastolic pressures were measured by a baumanometer (W.A. Baum, New York, NY) on the arm of a seated subject who had rested in a sitting position for 10 min before the measurement. Body fat mass and body fat percentage were measured by bioelectrical impedance analysis (Inbody 3·0; Biospace, Seoul, Korea). Waist-to-hip ratio (cm/cm) was determined by measurement of the circumference of waist and hip in the standing position. Waist circumference was measured to the nearest 0·1 cm at the level of the iliac crest by a tape while the subject was at minimal respiration. Hip circumference was measured at the level of the anterior superior iliac spine. Blood samples were drawn after an overnight fast and immediately centrifuged. Serum total cholesterol, triglycerides, high-density lipoprotein (HDL) cholesterol, low-density lipoprotein (LDL) cholesterol, uric acid and liver enzyme levels were determined by enzymatic methods with a chemistry analyser (TBA 200-FR, Toshiba, Japan). Plasma glucose was measured by the glucose oxidase method. High-sensitivity C-reactive protein (hsCRP) was determined by nephelometry (IMMAGE, Beckman Coulter, Fullerton, CA). Serum insulin was measured with an insulin radioimmunoassay kit (Biosource, Nivelles, Belgium), which had a reactivity of less than 0·2% to human proinsulin. Insulin resistance was estimated using the homeostasis model of assessment (HOMA), calculated as fasting glucose (mmol/l) × fasting insulin (mU/l)/22·5.^11^ Serum RBP4 concentrations were determined with an enzyme-linked immunosorbent assay using a commercial kit (AdipoGen, Seoul, Korea). The analysis used a RBP4 competitive ELISA system where 100 µg/l of recombinant human RBP4 expressed by an animal cell line (HEK293 cells) was coated onto a plate and varying concentrations of the recombinant RBP4 and a polyclonal antibody were added to the plate for a competitive reaction resulting in a standard curve. We performed a quantitative Western-equivalent analysis using a phosphoimage analyser (Fusifilm Co. LAS-1000, Tokyo, Japan) with the recombinant RBP4; its intra- and interassay coefficients of variation were 5·5% and 7·2%, respectively. We evaluated several serum samples during this study (*n* = 14) using Western blot analysis of RBP4. The RBP4 concentrations by Western blotting correlated with the results of the AdipoGen ELISA kits (*r* = 0·812, *P* < 0·001, *n* = 14).

A fatty liver was diagnosed on ultrasonography by a single experienced radiologist who was blinded to the laboratory data. Of the four known criteria used for the diagnosis of fatty liver (hepatorenal echo contrast, liver brightness, deep attenuation and vascular blurring), the participants were required to have hepatorenal echo contrast and liver brightness for the diagnosis of fatty liver.^12^ Subjects were categorized as the NAFLD group or the normal group based on the detection of a fatty liver by sonography.

### Statistical analyses

Data for anthropometric and metabolic characteristics of the study subjects were expressed as means ± SD or *n* (%). Variables that were skewed to the right were log-transformed for subsequent analysis. Differences between mean values of the NAFLD and normal groups were examined using the Student's *t*-test or χ^2^ test. The association of RBP4 with NAFLD was investigated using the multiple logistic regression analysis that was adjusted for age, gender, body mass index, waist circumference, body fat per cent, systolic and diastolic pressure, fasting glucose, HOMA, total cholesterol, triglycerides and HDL cholesterol. Pearson's correlation analysis was performed to establish the relationship between RBP4 and anthropometric, metabolic parameters and various liver enzymes in all subjects and then evaluated by gender. Statistical significance was accepted for *P*-values of < 0·05. All statistical analyses were performed using sas version 9·13 (SAS Institute, Cary, NC).

## Results

### Serum RBP4 levels in NAFLD

The characteristics of the study subjects are summarized in Table 1. The distribution of age and gender between the normal and the NAFLD groups was not different. Male participants comprised 58·1% of the normal group and 61·6% of the NAFLD group. The subjects in the NAFLD group had a greater weight, body mass index, waist circumference, waist-to-hip ratio, body fat mass, body fat per cent, systolic blood pressure, diastolic blood pressure, total cholesterol, triglycerides, LDL cholesterol, fasting glucose, fasting insulin, HOMA, HbA1c, uric acid, alanine aminotransferase (ALT), aspartate aminotransferase (AST), alkaline phosphatase (ALP), γ-glutamyltranspeptidase (GGT), hsCRP and RBP4 levels than control subjects. However, the HDL cholesterol was significantly lower in the NAFLD group. In all subjects, the mean serum RBP4 levels were 51·7 ± 14·6 mg/l in the normal group and 62·8 ± 16·0 mg/l in the NAFLD group (*P* < 0·0001). Male participants had higher levels of serum RBP4 than females (61·8 ± 14·7 mg/l *vs.* 49·3 ± 15·5 mg/l, *P* < 0·0001). For both genders, serum RBP4 levels in the NAFLD group were significantly higher than in the normal group (Table 1).

**Table 1:** Anthropometric and metabolic characteristics of the study subjects

	Total			Male§		Female§	
Variable	Normal (*n* = 86)	NAFLD (*n* = 73)	*P*-value†	Normal (*n* = 50)	NAFLD (*n* = 45)	Normal (*n* = 36)	NAFLD (*n* = 28)
Age (years)	49·7 ± 10·1	49·6 ± 12·5	0·9493	48·8 ± 10·9	47·3 ± 10·6	51 ± 8·9	53·4 ± 14·4
Gender, male (%)	50 (58·1)	45 (61·6)	0·6534				
Weight (kg)	63·9 ± 9·8	72·6 ± 9·2	< 0·0001	68·8 ± 8·7	77 ± 7·0***	57·2 ± 7·0	65·6 ± 8·1***
Body mass index (kg/m^2^)	23·7 ± 2·4	27 ± 2·3	< 0·0001	24 ± 2·2	26·6 ± 1·8***	23·2 ± 2·7	27·7 ± 2·8***
Waist circumference (cm)	80·2 ± 7·3	88·6 ± 5·8	< 0·0001	82·9 ± 6·1	89·1 ± 5·1***	76·4 ± 7·1	87·9 ± 6·9***
Waist-to-hip ratio (cm/cm)	0·86 ± 0·05	0·92 ± 0·06	< 0·0001	0·89 ± 0·04	0·92 ± 0·04***	0·83 ± 0·05	0·92 ± 0·08***
Body fat mass (kg)	14·8 ± 4·5	20·5 ± 5·0	< 0·0001	13·8 ± 3·8	18·3 ± 3·6***	16·3 ± 5·0	23·9 ± 5·2***
Body fat per cent (%)	23·2 ± 6·5	28·4 ± 7·4	< 0·0001	19·8 ± 4·4	23·7 ± 3·7***	27·8 ± 6·2	35·9 ± 5·3***
Systolic blood pressure (mmHg)‡	103 ± 1·1	109·7 ± 1·1	0·0027	103·9 ± 1·1	108·7 ± 1·1	101·8 ± 1·1	111·3 ± 1·2*
Diastolic blood pressure (mmHg)	66·1 ± 10·1	69·8 ± 10·4	0·0241	66·8 ± 10·0	68·9 ± 8·6	65·1 ± 10·3	71·3 ± 12·8*
Total cholesterol (mmol/l)	5·0 ± 0·8	5·4 ± 0·9	0·0045	5·0 ± 0·8	5·5 ± 0·7**	5·0 ± 0·9	5·4 ± 1·2
Triglyceride (mmol/l)‡	1·5 ± 1·7	2·2 ± 1·7	< 0·0001	1·8 ± 1·6	2·3 ± 1·6*	1·1 ± 1·6	2·0 ± 1·8***
HDL cholesterol (mmol/l)‡	1·2 ± 1·3	1·1 ± 1·2	< 0·0001	1·1 ± 1·2	1·0 ± 1·2***	1·4 ± 1·3	1·2 ± 1·2***
LDL cholesterol (mmol/l)	2·9 ± 0·6	3·2 ± 0·8	0·0150	2·9 ± 0·6	3·2 ± 0·6*	2·8 ± 0·7	3·0 ± 1·0
Fasting glucose (mmol/l)	5·1 ± 0·6	5·4 ± 0·6	0·0002	5·1 ± 0·4	5·4 ± 0·5**	5·1 ± 0·7	5·5 ± 0·6*
Fasting insulin (pmol/l)	54·8 ± 16·9	79·2 ± 27·1	< 0·0001	57·1 ± 19·4	76 ± 22·5***	51·6 ± 12·3	84·3 ± 32·8***
HOMA‡	1·6 ± 1·4	2·5 ± 1·5	< 0·0001	1·7 ± 1·4	2·4 ± 1·4***	1·5 ± 1·3	2·6 ± 1·6***
HbA1c (%)	5·6 ± 0·5	5·8 ± 0·4	0·0066	5·5 ± 0·4	5·7 ± 0·4**	5·7 ± 0·6	5·9 ± 0·4
Uric acid (µmol/l)	309 ± 89	363 ± 83	0·0005	363 ± 71	399 ± 77*	24·4 ± 59	303 ± 59***
ALT (IU/l)‡	15·4 ± 1·5	30·9 ± 1·7	< 0·0001	17·4 ± 1·4	36·6 ± 1·6***	13 ± 1·4	23·6 ± 1·6***
AST (IU/l)‡	19·4 ± 1·2	25·8 ± 1·4	< 0·0001	19·8 ± 1·2	27·7 ± 1·4***	18·9 ± 1·2	22·9 ± 1·4*
ALP (IU/l)‡	62·1 ± 1·3	71·2 ± 1·3	0·0019	65·5 ± 1·3	66·7 ± 1·3	57·8 ± 1·3	79 ± 1·4***
GGT(IU/l)‡	21·6 ± 1·7	41 ± 1·9	< 0·0001	28·4 ± 1·7	50·7 ± 1·9***	14·7 ± 1·4	29·1 ± 1·7***
hsCRP (mg/l)‡	0·06 ± 2·82	0·12 ± 2·54	< 0·0001	0·06 ± 2·63	0·09 ± 2·41*	0·05 ± 3·08	0·17 ± 2·42***
RBP4 (mg/l)	51·7 ± 14·6	62·8 ± 16·0	< 0·0001	58·7 ± 13·1	65·3 ± 15·8*	41·8 ± 10·4	58·9 ± 15·9***

† *P*-values are for differences between the NAFLD group and the control group using Student's *t*-test or the χ^2^ test (for gender) with total subjects.

‡ Geometric means and standard deviations are given. Logarithmic transformed values are used for comparing two groups.

§ Within each gender, statistical significance by Student's *t*-test was indicated by

* *P* < 0·05;

** *P* < 0·01;

*** *P* < 0·001.

Multiple logistic regression analysis was performed using NAFLD as a dependent variable. RBP4 was selected as a significant variable together with the body mass index, HOMA and HDL cholesterol level in all subjects (Table 2). We also performed multiple logistic regression analysis within each gender. In males, the RBP4 level {*P* = 0·0539; odds ratio (OR) [95% confidence interval (CI)]: 1·058 (0·999–1·120)} was a marginally significant variable together with total cholesterol [*P* = 0·0477, OR (95% CI): 1·542 (1·004–2·367)] and HDL cholesterol levels [*P* = 0·0031, OR (95% CI): 0·012 (< 0·001–0·226)]. By contrast, the RBP4 level was not significantly associated with NAFLD in females [*P* = 0·0873, OR (95% CI): 1·130 (0·982–1·299)].

**Table 2:** Multiple logistic regression analysis using NAFLD as a dependent variable with all subjects

	NAFLD			
	
Independent variables	Coefficient	SE	*P* value	OR(95% CI)
RBP4	0·0634	0·0222	0·0042	1·065 (1·020–1·113)
Age	0·0094	0·0330	0·7753	1·009 (0·946–1·077)
Gender*	1·5279	1·2262	0·2127	4·608 (0·417–50·96)
Body mass index	0·6519	0·2941	0·0266	1·919 (1·078–3·416)
Waist circumference	−0·0964	0·0828	0·2444	0·908 (0·772–1·068)
Body fat per cent	0·0432	0·0944	0·6473	1·044 (0·868–1·256)
Systolic blood pressure	0·0223	0·0388	0·5648	1·023 (0·948–1·103)
Diastolic blood pressure	−0·0362	0·0505	0·4725	0·964 (0·874–1·065)
Fasting glucose	−0·1860	0·6723	0·7820	0·830 (0·222–3·101)
HOMA	1·3235	0·4668	0·0046	3·757 (1·505–9·378)
Total cholesterol	0·5073	0·3921	0·1957	1·661 (0·770–3·581)
Triglycerides	0·2080	0·2658	0·4340	1·231 (0·731–2·073)
HDL cholesterol	−5·9610	1·7103	0·0005	0·003 (< 0·001–0·074)

SE, standard error; OR, odds ratio; CI, confidence interval.

* Reference group = male.

### Correlations between RBP4, metabolic parameters and liver enzymes

In all subjects (*n* = 159), RBP4 was found to be positively correlated with body mass index, waist circumference, waist-to-hip ratio, systolic and diastolic blood pressure, total cholesterol, triglycerides, LDL-cholesterol, fasting glucose, HOMA and uric acid levels, but not with body fat (Table 3). After adjusting for age, body mass index and the HOMA value, RBP4 still correlated with waist circumference, waist-to-hip ratio, diastolic pressure, triglycerides, fasting glucose and uric acid levels. The significant positive correlations between serum RBP4 and diastolic pressure, triglycerides and fasting glucose levels (*r* = 0·1726, *P* = 0·0415; *r* = 0·2304, *P* = 0·0062; *r* = 0·2706, *P* = 0·0012, respectively) were observed even after adjusting for age, gender, body mass index, HOMA and the presence of NAFLD. We also performed correlation analyses within each gender (Table 3). In males, diastolic blood pressure (*P* = 0·0136), fasting glucose (*P* = 0·0018) and HbA1c levels (*P* = 0·0062) were found to be positively associated with RBP4 after adjusting for age, body mass index and HOMA. In females, only triglyceride levels showed a significantly positive correlation (*P* = 0·0008) with RBP4 levels after adjusting for age, body mass index and HOMA.

**Table 3:** Simple correlation analyses between RBP4 levels and various parameters in all subjects and for each gender

	Simple correlation					
	
	All (*n* = 159)		Male (*n* = 95)		Female (*n* = 64)	
Parameters (*vs.* RBP4)	r	P	r	P	r	P
Age	0·1096	0·1705	0·1347	0·1955	0·2668	0·0331
Body mass index	0·2174	0·0061	0·1103	0·2900	0·3554	0·0040
Waist circumference	0·3657	< 0·0001	0·2104	0·0418	0·3841	0·0017
Waist-to-hip ratio	0·3814	< 0·0001	0·2130	0·0393	0·4032	0·0010
Body fat mass	0·0796	0·3200	0·1155	0·2676	0·3548	0·0040
Body fat per cent	−0·0719	0·3695	0·1212	0·2445	0·3638	0·0031
Systolic blood pressure*	0·2398	0·0024	0·2589	0·0117	0·2523	0·0443
Diastolic blood pressure	0·2404	0·0024	0·2784	0·0066	0·2420	0·0540
Total cholesterol	0·2194	0·0056	0·0984	0·3456	0·3630	0·0032
Triglycerides*	0·4235	< 0·0001	0·1698	0·1017	0·5507	< 0·0001
HDL-cholesterol*	−0·1306	0·1019	0·1477	0·1555	−0·0844	0·5076
LDL-cholesterol	0·1769	0·0262	0·0311	0·7658	0·2638	0·0352
Fasting glucose	0·2557	0·0012	0·2539	0·0135	0·3117	0·0122
Fasting insulin	0·1182	0·1511	−0·0607	0·5741	0·3344	0·0084
HOMA*	0·1930	0·0184	0·0266	0·8061	0·3804	0·0025
HbA1c	0·1558	0·0521	0·2348	0·0235	0·2742	0·0297
Uric acid	0·4089	< 0·0001	0·1690	0·1035	0·3786	0·0020
ALT*	0·3599	< 0·0001	0·1803	0·0854	0·4062	0·0009
AST*	0·2428	0·0021	0·0995	0·3399	0·3313	0·0075
ALP*	0·0602	0·4526	−0·1253	0·2289	0·2496	0·0467
GGT*	0·4961	< 0·0001	0·3399	0·0008	0·4851	< 0·0001
hsCRP*	0·0194	0·8090	−0·1117	0·2837	0·2165	0·0857

* Logarithmic transformed values are used.

**Fig. 1:**
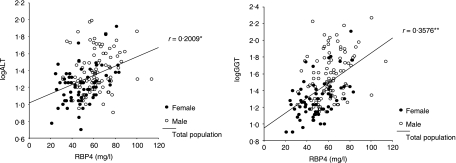
Serum RBP4 levels were positively correlated with log-transformed ALT and GGT after controlling for age, gender, body mass index and HOMA (*P = 0·0169, **P < 0·0001 in all subjects).

Interestingly, serum RBP4 levels were positively correlated with serum ALT, AST and GGT levels (Table 3). After adjusting for age, gender, body mass index and HOMA, serum ALT and GGT levels were positively associated with serum RBP4 levels (*r* = 0·2009, *P* = 0·0169; *r* = 0·3576, *P* < 0·0001, respectively, Fig. 1). Moreover, the significant correlations between serum RBP4 and GGT levels were maintained even after adjusting for age, gender, body mass index, HOMA and the presence of NAFLD (*r* = 0·3097, *P* = 0·0002). When we performed the correlation analysis adjusted for age, gender, HOMA and waist circumference as a marker of abdominal obesity, there were significant correlations between the serum RBP4 levels and the serum ALT and GGT levels (*r* = 0·2004, *P* = 0·0172; *r* = 0·3588, *P* < 0·0001, respectively).

## Discussion

RBP4, a recently identified adipokine, may contribute to systemic insulin resistance.^9^ It is well known that NAFLD is associated with insulin resistance. The present study demonstrated that serum RBP4 concentrations are elevated in nondiabetic subjects with NAFLD compared to normal healthy controls, and that circulating RBP4 was an independent factor associated with NAFLD. In addition, there were positive correlations between RBP4 and liver enzymes. Serum RBP4 levels correlated with several parameters related to insulin resistance including waist circumference, HOMA index, fasting glucose and triglycerides levels. Unfortunately, we could not assess the causal relationship between NAFLD and RBP4 levels because the present study was designed as a cross-sectional study.

Several previous reports including a study of Korean subjects^13^ have shown that circulating RBP4 levels correlate with body mass index,^10^,^14^ waist circumference^13^ and insulin resistance.^10^,^13^ Similarly, we found that serum RBP4 levels positively correlated with the insulin resistance index. The subjects with NAFLD were more obese and more insulin resistant in our study. Accordingly, obesity and increased insulin resistance, in subjects with NAFLD, may contribute to elevation of the circulating RBP4 levels. However, logistic regression analysis revealed that the RBP4 level was an independent factor associated with NAFLD. In addition, the results of the correlation analysis showed that the serum RBP4 level positively correlated with ALT and GGT levels even after adjusting for age, gender, body mass index and HOMA. The positive correlations between serum RBP4 and GGT levels were even more prominent in subjects with NAFLD than in controls after adjusting for age, gender, body mass index and HOMA (NAFLD subjects: *r* = 0·3478, *P* = 0·0049 *vs.* controls: *r* = 0·2048, *P* = 0·0821). These results suggest that a high RBP4 level likely indicates a predictor for NAFLD.

It is not yet known whether RBP4 affects lipid metabolism in hepatocytes. A possible explanation is that RBP4 may influence the transactivation of retinoic acid-sensitive transcription factors such as retinoic acid receptor (RAR) and retinoic acid-X receptor (RXR) in NAFLD. RXRs bind to DNA as obligate heterodimers with peroxisome-proliferator activated receptors (PPARs) that regulate the transcription of genes involved in fatty acid metabolism.^15^ Another possible mechanism explaining the link between RBP4 and NAFLD is that changes in retinoid metabolism induced by RBP4 might alter the tissue level of retinol. A previous study reported that normalization of circulating RBP4 by synthetic retinoid improves insulin resistance.^9^

In rodents, it has been reported that RBP4, normally expressed in liver and hepatocytes, is the principal source of circulating RBP4 under normal conditions and adipose tissue is the organ expressing the second highest level of RBP4·^16^ Recently, Janke *et al*. found no significant relationship between adipose tissue RBP4 expression and serum RBP4 levels in postmenopausal women,^17^ and Stefan *et al*. showed that circulating RBP4 was not associated with the amount of visceral and subcutaneous abdominal fat.^18^ The above findings suggest that adipose tissue is likely to be a less important source of circulating RBP4 in humans than in animals. At present, it is unclear whether the liver is the major source of circulating RBP4 in humans and which organ is more responsible for the increase of circulating RBP4 in NAFLD.

In the multiple logistic regression analysis, the association between NAFLD and RBP4 was observed in all subjects. However, when we analysed within each gender, the RBP4 level was not a significant factor associated with NAFLD in females, and was a marginally significant variable in males. Serum RBP4 levels were higher in male subjects than in females, which is consistent with another Korean study.^13^ Our study subjects included pre- and postmenopausal women (24 pre- and 40 postmenopausal women). Both pre- and postmenopausal women had significantly lower serum RBP4 levels than men (data not shown). Further study is needed to elucidate whether this gender difference is due to the small sample size, especially for females, or if the relationship between the RBP4 level and NAFLD is actually gender specific.

Limitations of this study include the following. First, we defined NAFLD as a fatty liver based on ultrasonography without histological confirmation. However, we consider ultrasonography to be a reasonable alternative to liver biopsy in relatively healthy subjects. According to a recent review, ultrasound scanning, when positive, can provide a high degree of diagnostic certainty, depending on the prevalence of fatty liver in the population being studied.^19^ A recent study reported that circulating RBP4 was positively associated with liver fat content quantified by magnetic resonance (MR) spectroscopy. This result is consistent with the findings of our study.^18^ The second limitation is, as mentioned earlier, that the causal relationship between NAFLD and the RBP4 level could not be determined because of the cross-sectional nature of the present study. In conclusion, serum RBP4 levels are significantly associated with NAFLD and various liver enzymes. These findings suggest that this newly defined adipokine might be related to pathogenesis of NAFLD.
